# Analytical Design and Polyphase Implementation Technique for 2D Digital FIR Differentiators

**DOI:** 10.3390/s24237870

**Published:** 2024-12-09

**Authors:** Radu Matei, Doru Florin Chiper

**Affiliations:** 1 Faculty of Electronics, Telecommunications and Information Technology, Gheorghe Asachi Technical University of Iaşi, 700506 Iaşi, Romania; chiper@etti.tuiasi.ro; 2 Institute of Computer Science, Romanian Academy—Iaşi Branch, 700481 Iaşi, Romania; 3 Technical Sciences Academy of Romania—ASTR, 700050 Iaşi, Romania; 4 Academy of Romanian Scientists—AOSR, 030167 Bucharest, Romania

**Keywords:** approximations, frequency transformations, 2D FIR filters, differentiators

## Abstract

In this work, an analytical method in the frequency domain is proposed for the design of two-dimensional digital FIR differentiators. This technique uses an approximation based on two methods: the Chebyshev series and the Fourier series, which, finally, lead to a trigonometric polynomial, which is a remarkably precise approximation of the transfer function of the ideal differentiator. The digital differentiator is applied to three test images, one greyscale image and two binary images, and simulation results show its performance in the processing task. Also, based on the fact that this 2D differentiator is separable on the two frequency axes, we propose an efficient implementation at the system level, using polyphase filtering. The designed digital differentiator is very accurate and efficient, having a high level of parallelism and reduced computational complexity.

## 1. Introduction

The field of two-dimensional digital filters [1,2] has been continuously evolving, being a fundamental topic of research in digital image processing [3,4]. The analytic design techniques commonly employed for 2D FIR and IIR filters generally start from 1D prototypes, either digital or analog, with specified parameters. Various frequency transformations are then applied to the chosen prototype, which leads to a 2D filter with a required frequency response. Several early papers like [5] and relatively more recent papers like [6,7,8,9] have elaborated and refined these analytic design methods for 2D filters. A wide variety of 2D filters exist, each with a particular shape of the frequency response, such as circular or elliptical, fan or square shaped, directional filters, etc. Some design techniques for 2D IIR filters having a separable denominator were elaborated in [10,11,12]. Several papers approach various directional 2D filters applied in edge detection [13], separable filters for directional smoothing [14], efficient directional Gaussian smoothing [15], etc.

Edge detection is a complex, essential task in the image processing field, consisting in the process of locating, detecting and, eventually, extracting object contours and, in general, sharp discontinuities in images. Most edge-detection techniques usually belong to one of two classes: the gradient method, which detects edges by searching for maxima and minima in the first derivative of the image, and the Laplacian method, which finds zero crossings in the second derivative of the image. The most commonly used edge-detection techniques are the Sobel, Prewitt, Roberts, Canny, Frei-Chen and Laplacian operators. Some comprehensive, comparative studies of various edge-detection methods and their performance can be found in works such as [16,17]. In [18], the performance of various edge detectors is evaluated by taking into account the peak signal-to-noise ratio. In [19], a fractional differentiation is employed for edge detection.

Regarding discrete differentiation, the early paper [20] proposes several finite-difference schemes with an improved representation of the scale range (spectral resolution) for the evaluation of first, second and higher order derivatives, which have examples of application in fluid mechanics but are also useful in image processing. Then, the work [21] presents the theoretical background for generating FIR masks for signal and image differentiation of any order, as well as a very efficient algorithm for implementing the implicit discrete differentiation of signals and images. The advantages of using implicit finite differences for the fast, precise and reliable differentiation of multidimensional signals on rectangular grids are investigated in the paper [22].

Many researchers have approached 1D and 2D differentiators, owing to their utility in signal and image processing tasks. Several design procedures and applications of digital differentiators, including edge detection, are studied in [23]. In [24,25], FIR differentiators are designed using numerical optimization techniques to derive the system coefficients, using the L1 norm to obtain the 2D differentiator with minimum L1 error. In [26], a heuristic method using the cuckoo-search algorithm is adopted to achieve the optimal design of a 2D differentiator, also based on the L1 norm.

A design procedure is proposed in [27] for 2D maximally linear FIR differentiators relying on Taylor series. A similar method is also applied in [28] for higher-degree FIR differentiators. In [29], FIR-type approximations of IIR differentiators are given, with applications in image edge detection. In [30], 2D differentiators are used to design 2D variable fractional delay FIR filters. Paper [31] presents a family of band-limited differentiators using a polynomial estimation with least squares. A transfer function expression for the maximally flat low-pass differentiator of FIR type is determined in [32], while FIR band-pass digital differentiators having the characteristics of a flat passband and equiripple stopband are described in [33].

Apart from the papers mentioned before, which approach the design of 2D IIR filters with a separable denominator, various procedures have been elaborated for the efficient design of other separable 2D filters, which are very convenient for implementation. For instance, some architectures using separable filter structures for general 2D FIR filtering are given in [34]. Circularly symmetric 2D filters based on separable 1D transfer functions are proposed in [35]. Some essentially separable FIR filters using McClellan transformation are described in [36]. A design approach for a separable 2D FIR filter with sparse coefficients is elaborated in [37].

Two-dimensional differentiators are extensively used in image processing tasks, in a vast range of applications, in particular, computer vision, surveillance and remote sensing, in which the essential component is the image sensor. The differentiator can fulfil pre-processing tasks, like edge detection, in a more complex processing of images acquired by sensors.

An analytical design procedure for a 2D IIR differentiator has been developed in [38], using a Chebyshev–Padé approximation approach. A circular 2D FIR filter bank in both uniform and non-uniform version is designed analytically in [39] and an efficient implementation is also proposed, using polyphase decomposition and also block filtering technique, yielding a filter with a high level of parallelism and low computational complexity. In [40], a broader class of 2D IIR filters with orientation-selective frequency response is designed using an analytical approach, and some useful applications are given, for instance, detecting straight lines with a given orientation in synthetic or real-life images.

Regarding specific implementation techniques for separable FIR filters, a pipeline architecture for VLSI implementation is proposed in [41], while energy, performance and precision are analyzed in the work [42] for FPGA and GPU implementations.

In this paper, an analytical design technique is elaborated for FIR 2D digital differentiators, in the frequency domain, relying on a precise approximation of the 1D differentiator, using Fourier series and Chebyshev series, without employing any complicated numerical optimization algorithms.

A significant advantage is that the proposed 2D FIR differentiator is separable, its frequency response resulting as a product of the responses of two 1D differentiators, along the two frequency plane axes. Unlike most approaches to this topic in the literature, the design method proposed here is rather analytical, using only accurate approximations such as Chebyshev series, without resorting to global optimization techniques.

This work is organized as follows: the analytical design method is discussed in Section 2, with two types of approximation being approached in separate sub-sections. The simulation results of the designed FIR differentiator, applied to some binary and also grayscale images, are given in Section 3, highlighting its capabilities in a simple edge-detection task. Section 4 approaches an efficient implementation using the polyphase approach and leading to a 2D differentiator structure with a high level of parallelism and a low arithmetic complexity. Discussions regarding the proposed design and performance of the resulted 2D differentiator are considered in Section 5, followed by concluding remarks; ideas for continuing research on this topic in future work are given in the final section.

## 2. Analytical Design Method for the FIR Differentiator

The analytical design method elaborated here is rather straightforward and easy to use. The starting point is the expression of the 2D differentiator transfer function, in the two frequency variables $\omega_{1}$ and $\omega_{2}$:

(1) $$H_{2D}(\omega_{1},\omega_{2})=-\omega_{1}\cdot \omega_{2}$$

This equation is formulated as the product of transfer functions of 1D differentiators, each operating on one of the two frequency axes, $H_{1D}(\omega_{1})=j\omega_{1}=s_{1}$ and $H_{1D}(\omega_{2})=j\omega_{2}=s_{2}$. Here, $s_{1}$ and $s_{2}$ are the complex frequencies (written as Laplace variables) on the two frequency axes, in the same way that H(s)=s is the transfer function of a differentiator acting in the continuous time domain. Therefore, the 2D differentiator frequency response has the following separable form:

(2) $$H_{2D}(\omega_{1},\omega_{2})=H_{1D}(\omega_{1})\cdot H_{1D}(\omega_{2})$$

This implies that the design of a FIR 2D differentiator reduces, in fact, to designing a 1D differentiator, which requires finding an approximation as accurate as possible for the linear function H(ω)=ω. The design of the differentiator will be presented in two versions. As shows (1), the differentiator frequency response results as product of the two frequency variables $\omega_{1}$ and $\omega_{2}$. In order to derive a FIR differentiator, we will search for a trigonometric polynomial approximation of the real linear function H(ω)=ω, using two different approaches: the Fourier series and the Chebyshev series approximations.

### 2.1. Approximation of the 1D FIR Differentiator Using Fourier Series

As mentioned previously, we have to find a convenient trigonometric polynomial approximation of the linear function H(ω)=ω on the frequency interval ω∈[−π,π]. The natural, easiest approach to obtain such an approximation would be to consider the linear function as a periodic function on the frequency axis, with a period 2π, and then to calculate analytically its Fourier series. Since the function crosses the origin and has odd parity, we expect to obtain a Fourier series in sine terms.

**Figure 1:**
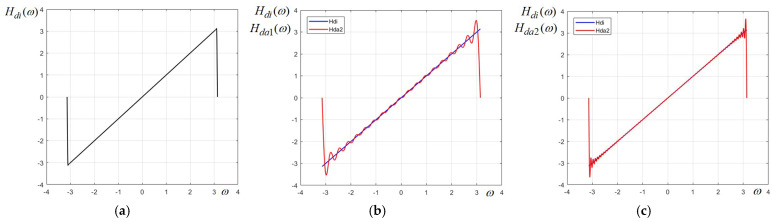
(**a**) Ideal prototype function; approximation using Fourier series truncated at (**b**) 18 terms; (**c**) 72 terms.

Considering the linear function H(ω)=ω on the range ω∈[−π,π] as a “generating pulse” (displayed in Figure 1a, where the abrupt transitions at the margins of the frequency interval are also shown), using either the complex form or, directly, the real (harmonic) form of the Fourier series, we easily derive the following approximation:

(3) $$\begin{array}{l} H_{D1}\left(\omega \right)≅H_{DA1}\left(\omega \right)=\sum_{n=1}^{N}a_{n}\cdot \sin n\omega =-\sum_{n=1}^{N}\left(\frac{2}{n}\right)\cdot \cos(n\pi )\cdot \sin n\omega =\sum_{n=1}^{N}\left(\frac{2}{n}\right)\cdot {\left(-1\right)}^{n+1}\cdot \sin n\omega \\ =2\sin\omega -\sin2\omega +(2/3)\cdot sin3\omega -0.5\cdot sin4\omega +0.4\cdot sin5\omega -(1/3)\cdot sin6\omega +\ldots \end{array}$$

In essence, this is the series of a well-known signal that is called the ramp or saw-tooth signal. The linear function H(ω)=ω corresponding to a 1D differentiator can, thus, be approximated by truncating the Fourier series given by (3) to a specified order, which gives an acceptable error. Let us first consider the approximation $H_{da1}(\omega )$, resulted by keeping only the first N=18 terms of the Fourier series. The approximation $H_{da1}(\omega )$ is plotted in red in Figure 1b, while the ideal function $H_{di}(\omega )=\omega$ is plotted in blue. We notice that, even if the approximation order is relatively high, the function $H_{da1}(\omega )$ has a significant ripple near the limits of frequency interval [−π,π]. This is the well-known Gibbs phenomenon, which in this case, arises due to the abrupt transition of the prototype function represented in Figure 1a. If we increase the order of approximation four times, taking N=72, the oscillations decrease in amplitude and increase in frequency, but they do not disappear completely. This approximation $H_{da2}(\omega )$ is plotted in red in Figure 1c.

**Figure 2:**
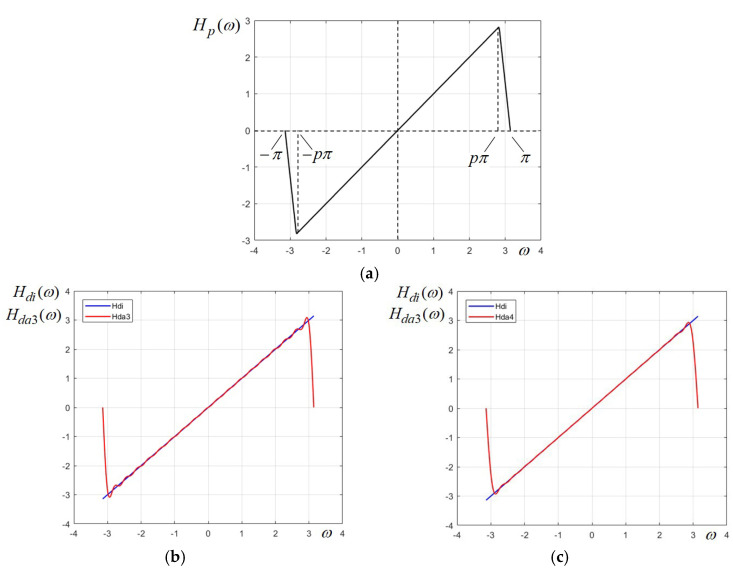
(**a**) Differentiator prototype with finite slopes at the margins. (**b**,**c**) Approximations with 18 terms of the Fourier series, with values of parameter *p*: (**b**) *p* = 0.96; (**c**) *p* = 0.94.

In order to overcome this issue and to be able to efficiently use the Fourier series approach, we consider the prototype function $H_{p}(\omega )$ in Figure 2a, which no longer has a steep transition at the margins of the frequency interval [−π,π], but linear portions with a specified slope. The frequency values corresponding to the breaking points of this piece-wise linear function are at −pπ and pπ, where p is a value less than 1. We will see that such a function can be more smoothly and efficiently approximated, due to the fact that the linear portion does no longer terminate abruptly, as in the previous case. Considering again this piece-wise linear function on the frequency range ω∈[−π,π] as a “generating pulse”, we derive the following expansion in Fourier series:

(4) $$H_{D1}\left(\omega \right)≅H_{DA2}\left(\omega \right)=\sum_{n=1}^{N}a_{n}\cdot \sin n\omega =\frac{2}{(1-p)\pi }\cdot \sum_{n=1}^{N}\frac{1}{n^{2}}\cdot \left(\sin(np\pi )-p\cdot \sin(n\pi )\right)\cdot \sin n\omega$$

Calculating the Fourier series coefficients with p = 0.94, and truncating it to the first 18 terms, then factoring the trigonometric polynomial, the following approximation results:

(5) $$\begin{array}{l} H(\omega )=\omega ≅H_{P}(\omega )=4.169769\cdot \sin\omega \cdot \left(cos\omega +1.237361\right)\\ \cdot \left(\cos2\omega +3.774427\cdot \cos\omega +2.810316\right)\cdot \left(\cos2\omega +2.895772\cdot \cos\omega +2.156942\right)\\ \cdot \left(\cos2\omega +1.656776\cdot \cos\omega +1.530918\right)\cdot \left(\cos2\omega +0.223983\cdot \cos\omega +1.230923\right)\\ \cdot \left(\cos2\omega -1.227829\cdot \cos\omega +1.389549\right)\cdot \left(\cos2\omega -2.523932\cdot \cos\omega +1.927744\right)\\ \cdot \left(\cos2\omega -3.507092\cdot \cos\omega +2.590973\right)\cdot \left(\cos2\omega -4.049879\cdot \cos\omega +3.056218\right) \end{array}$$

which is represented graphically in Figure 2c. For the most part, the approximation function is perfectly linear, with some negligible undulations towards the margins of the interval.

### 2.2. Approximation of the 1D FIR Differentiator Using Chebyshev Series

In this section, we derive a trigonometric polynomial approximation of the linear function H(ω)=ω on the frequency range [−π,π] using another approach. The approximation of the linear function H(ω)=ω is more conveniently found indirectly, by deriving, first, a trigonometric polynomial approximation of the function $H_{1}(\omega )=\omega /\sin\omega$. Since this is an even function, we should be able to find a polynomial of a certain order in variable cosω, which approximates $H_{1}(\omega )$ with a specified precision. In order to achieve this, we will, first, make the following change in variable [37]:

(6) $$\omega =\arccos\left(x/\pi \right)↔x=\pi \cos\omega$$

Substituting ω=arccos(x/π) in $H_{1}(\omega )=\omega /\sin\omega$, the following function G(x) results:

(7) $$G(x)=\frac{\arccos\left(x/\pi \right)}{\sin\left(\arccos\left(x/\pi \right)\right)}=\frac{\arccos\left(x/\pi \right)}{\sqrt{1-{\left(x/\pi \right)}^{2}}}$$

Next, using MAPLE or another symbolic computation software, we find a Chebyshev series approximation of the function G(x), which is a polynomial $G_{P}(x)$ in variable *x*, of a certain order depending on required precision. For instance, a very accurate approximation is the following polynomial of order 18, in the factored form:

(8) $$\begin{array}{l} G_{P}(x)=0.539833\cdot 10^{-5}\cdot (x+3.229409)\cdot (x^{2}+5.646051\cdot x+8.159765)\\ \cdot (x^{2}+4.256689\cdot x+5.107755)\cdot (x^{2}+2.348221\cdot x+2.338462)\cdot (x^{2}+0.151914\cdot x+1.139965)\\ \cdot (x^{2}-2.069794\cdot x+2.082126)\cdot (x^{2}-4.051382\cdot x+4.764269)\cdot (x^{2}-5.553138\cdot x+7.979133)\\ \cdot (x^{2}-6.380594\cdot x+10.208594) \end{array}$$

In (8), *x* is, therefore, an intermediate variable, used to derive the required approximation. The following step is to substitute back in all the factors from (8) and the variable x=πcosω from (6), and using the trigonometric identity ${\left(\cos x\right)}^{2}=0.5\cdot \cos2x+0.5$, the following factored trigonometric approximation $H_{P}(\omega )$ for linear function H(ω)=ω is found:

(9) $$\begin{array}{l} H(\omega )=\omega ≅H_{P}(\omega )=1513.7833\cdot \left(\sin\omega \right)\cdot \left(\cos\omega +1.028477\right)\\ \cdot \left(\cos2\omega +1.7981022\cdot \cos\omega +0.827593\right)\cdot \left(\cos2\omega +1.355634\cdot \cos\omega +0.518049\right)\\ \cdot \left(\cos2\omega +0.747841\cdot \cos\omega +0.237176\right)\cdot \left(\cos2\omega +0.04838\cdot \cos\omega +0.115619\right)\\ \cdot \left(\cos2\omega -0.65917\cdot \cos\omega +0.2111775\right)\cdot (\cos2\omega -1.290249\cdot \cos\omega +0.483211)\\ \cdot (\cos2\omega -1.768515\cdot \cos\omega +0.809275)\cdot (\cos2\omega -2.032036\cdot \cos\omega +1.035396) \end{array}$$

Using, in (9), the identities $\cos\omega =0.5\cdot (z+z^{-1})$ and $\sin\omega =\left(z-z^{-1}\right)/2j=-0.5j\cdot (z-z^{-1})$, where *z* is the complex frequency variable z=exp(jω), the frequency response $H_{P}(\omega )$ of the FIR differentiator can be also expressed as a transfer function $H_{FIR}(z)$, which is a polynomial in variable *z* (where the constant in front is β=−0.00577463 and M=8):

(10) $$H_{FIR}(z)=\sum_{n=-M}^{M}a_{n}\cdot z^{n}=\beta \cdot j\cdot \left(z-z^{-1}\right)\cdot \left(z+z^{-1}+c_{0}\right)\cdot \prod_{i=1}^{M}\left(z^{2}+b_{i}z+c_{i}+b_{i}z^{-1}+z^{-2}\right)$$

The coefficient values $b_{i}$ and $c_{i}$ of each factor in (10) are easily identified from the corresponding factor of the frequency response (9).

The polynomial $H_{FIR}(z)$ has symmetric terms with positive and also negative powers of *z* and its coefficients are anti-symmetric, such that $a_{-n}=-a_{n}$. The degree of the polynomial, in our case, as results from (10), is N=2(M+1)=18 and, thus, the polynomial $H_{FIR}(z)$ has, in total, 37 coefficients. These coefficients of the derived FIR differentiator are given in Table 1, from the maximum order N=18 to N=−18.

**Figure 3:**
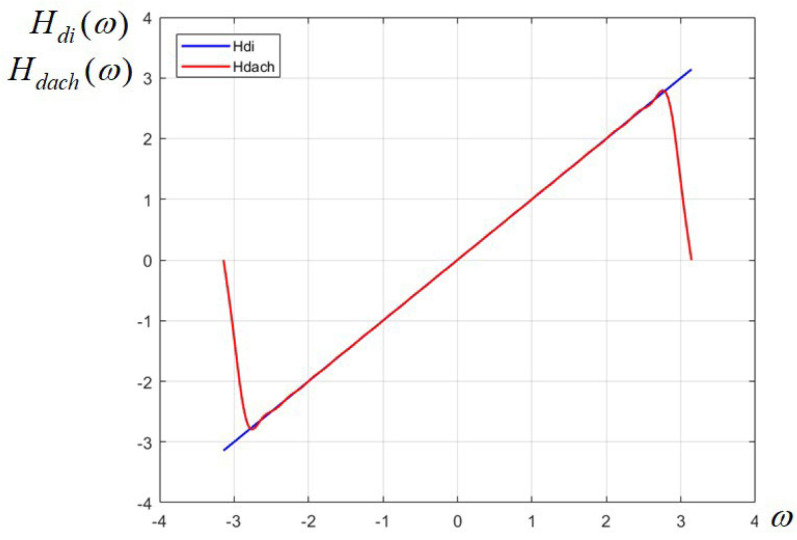
Frequency response of the 1D FIR differentiator (in red) designed with Chebyshev series and the linear function (in blue).

The coefficients corresponding to negative powers are anti-symmetric, with changed sign (for instance, $a_{4}=0.1892063$, $a_{-4}=-0.1892063$ etc.). The polynomial approximation $H_{P}(\omega )$ is plotted in Figure 3, superposed with the linear function H(ω)=ω. We notice that the approximation is very accurate on the frequency interval [−π,π] and it diverges only very close to the margins of the interval, due the periodicity of the cosine function. Finally, the transfer function of the overall 2D FIR differentiator has the matrix form

(11) $$H_{2DFIR}(z_{1},z_{2})=H_{FIR}(z_{1})\cdot H_{FIR}(z_{2})=\lambda \cdot z_{1}\times \left(R^{T}⊗R\right)\times z_{2}^{T}$$

where $R^{T}⊗R$ is a 37×37 matrix resulted as the outer product ⊗ (2D convolution) of the 1×18 vector R with its transpose $R^{T}$. The vector **R** is, in fact, the impulse response of the 1D FIR differentiator whose element values are given in Table 1. The vectors $z_{1}$ and $z_{2}$ are $z_{1}=\left[\begin{array}{ccccc} z_{1}^{18} & z_{1}^{17} & \ldots & z_{1}^{-17} & z_{1}^{-18} \end{array}\right]$, $z_{2}=\left[\begin{array}{ccccc} z_{2}^{18} & z_{2}^{17} & \ldots & z_{2}^{-17} & z_{2}^{-18} \end{array}\right]$ and the constant in front is $\lambda ={\left(\beta \cdot j\right)}^{2}=-0.333464\cdot 10^{-4}$.

**Figure 4:**
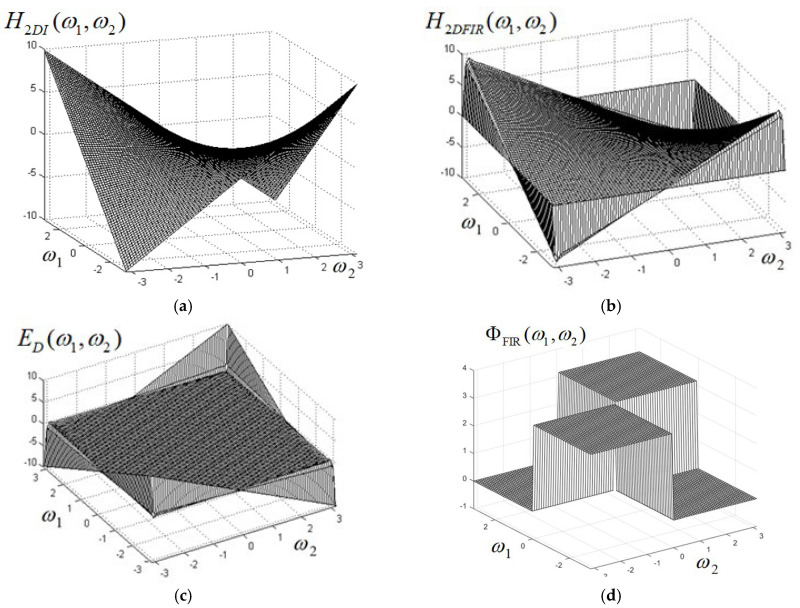
(**a**) Frequency response of the ideal 2D differentiator; (**b**) frequency response of the designed FIR 2D differentiator; (**c**) error surface function between the FIR and ideal differentiators; (**d**) phase of the designed 2D FIR differentiator.

The frequency response of the 2D differentiator in the ideal version $H_{2DI}(\omega_{1},\omega_{2})$ given by expression (1) is represented graphically in Figure 4a, while the frequency response $H_{2D\mathrm{FIR}}(\omega_{1},\omega_{2})$ of the designed 2D FIR differentiator is shown in Figure 4b. The shape is very accurate through the entire frequency plane, except near the margins, where the function varies rapidly to zero, due to periodicity on the frequency axes. The difference between the frequency responses of the designed 2D FIR differentiator and of the ideal 2D differentiator is displayed in Figure 4c. This can be considered as an error function and it can be noticed that it is practically zero all over the frequency plane, except the region very close to the margins, which does not affect the differentiator operation; therefore, the proposed method gives a very accurate digital differentiator.

The phase characteristic $\Phi_{\mathrm{FIR}}(\omega_{1},\omega_{2})$ of the designed FIR differentiator is shown in Figure 4d, and it is practically similar to the phase of the ideal 2D differentiator. Since $\Phi_{\mathrm{FIR}}(\omega_{1},\omega_{2})$ is a real function, even if, near the margins of the interval (−π,π), the function varies rapidly to zero, the phase preserves the correct value within this range.

## 3. Simulation Results

**Figure 5:**
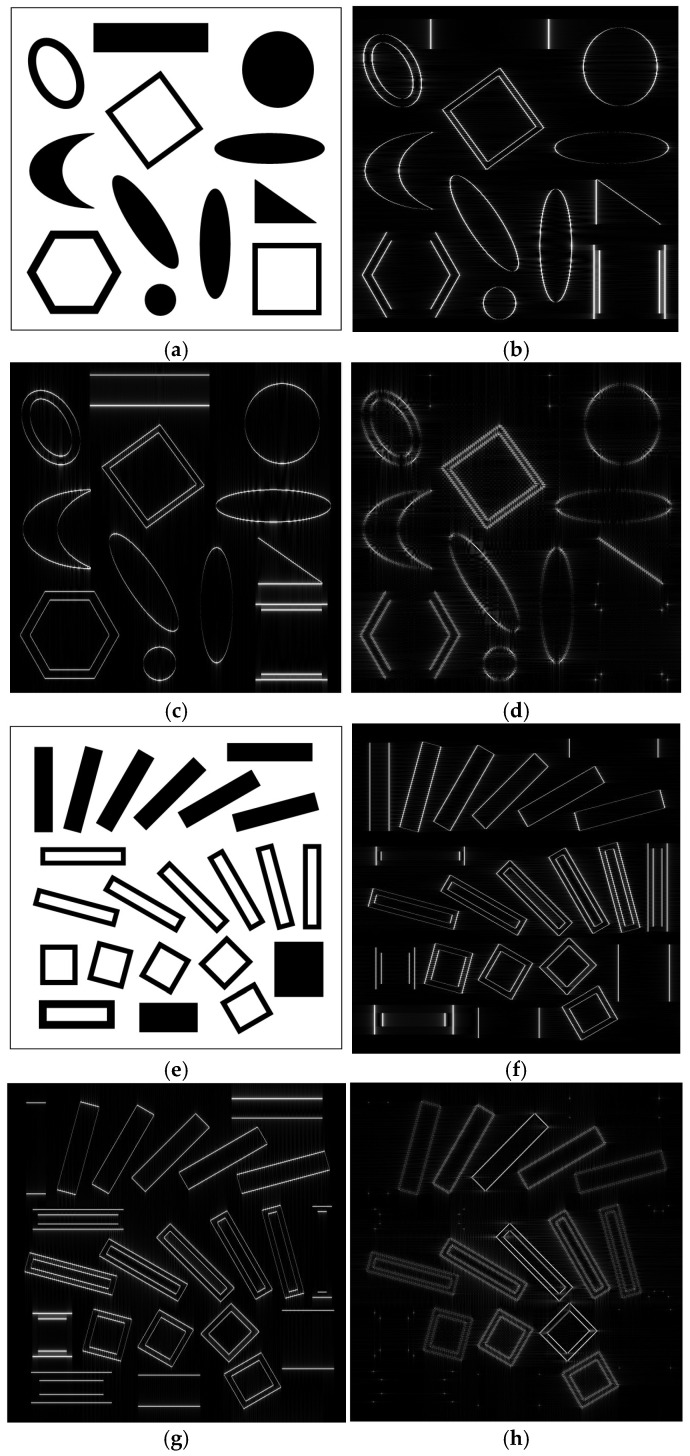
(**a**,**e**) Binary test images; (**b**,**c**,**f**,**g**) output images resulted from the 1D FIR differentiator, applied along rows and columns of the image; (**d**,**h**) output images resulted from the 2D FIR differentiator.

In this section, in order to assess the performance of the obtained differentiator, it is applied to three test images, namely, two synthetic binary images and one real-life greyscale image. The differentiator achieves an elementary edge-detection task, as show the results of the simulations. The differentiator is first tested on the binary image in Figure 5a, featuring some simple geometrical black objects against a white background. The images in Figure 5b,c have resulted by applying the 1D FIR differentiator described by (9) to the rows and columns of the image, respectively. The image in Figure 5d is yielded by applying the 2D FIR digital differentiator. We observe in Figure 5d that the edges of rectangular objects parallel with the axes are not detected, while the edges of curved objects (i.e., circles, ellipses) are all more or less visible.

The second test image (Figure 5e) is also a binary image, this time containing only rectangular objects, oriented at various angles with respect to the axes. As should be expected, the 1D differentiators detect the edges of the objects, along the two axes (shown in Figure 5f,g). If both 1D differentiators are applied sequentially, which is equivalent to applying a 2D differentiator, the image in Figure 5h results, in which the detected edges are more or less visible, depending on their orientation. We notice that objects oriented at 45 degrees (a square in (d), and a square and two rectangles in (h)) are most visible, while objects with orientation angles closer to 0 and 90 degrees are barely visible. The rectangles oriented horizontally and vertically (parallel to the axes) are not detected at all; their sides are erased and only the corners are detected.

**Figure 6:**
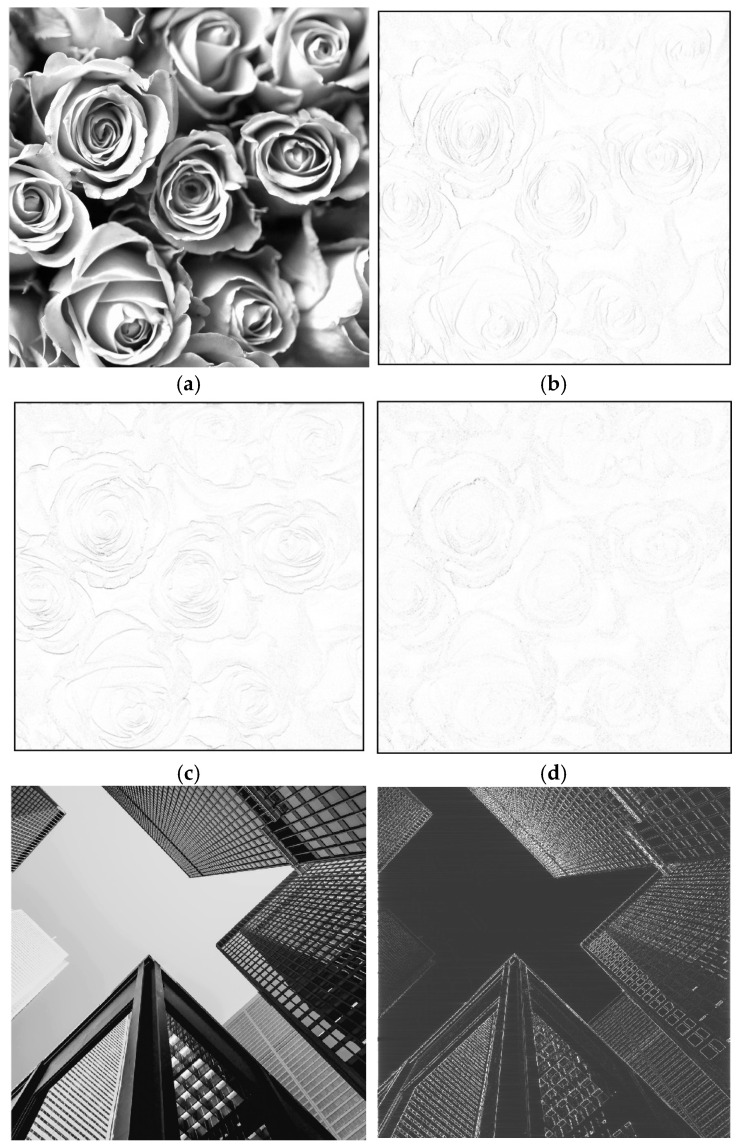

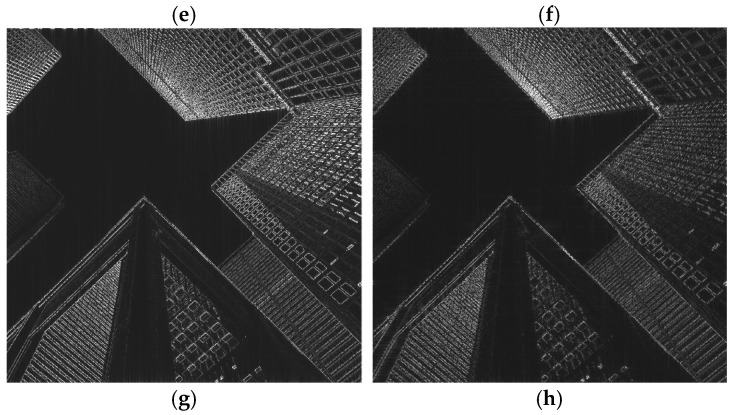
(**a**) Greyscale image “Roses” of size 2667 × 2667; (**e**) greyscale image “Skyscrapers” of size 999 × 999; (**b**,**c**,**f**,**g**) output images resulted from the 1D FIR differentiator, applied on rows and columns, respectively; (**d**,**h**) output images resulted from the 2D FIR differentiator.

The next simulations have been performed on four real-life grayscale images which contain coarse and fine details and can demonstrate the operation of the partial (1D) differentiators and the overall (2D) differentiator. The first test image is shown in Figure 6a, representing some roses. Applying the 1D differentiator along the two axes, the images in Figure 6b,c result. Applying the 2D FIR differentiator, the image displayed in Figure 6d results, showing the detected edges, as black contours on a white background. In the following examples, however, the output images are displayed how they result from the simulations, namely, as white contours on a black background.

The second test image “Skyscrapers” of size 999 × 999 is given in Figure 6e and shows some tall buildings viewed from the ground level. Due to perspective effect, the image features straight lines oriented along various directions and also progressive coarse to fine details, as higher floors details appear smaller and closer to each other. Figure 6f,g show the output images resulted from the 1D FIR differentiator, applied on rows and columns, respectively; (h) is the output image resulted from the 2D FIR differentiator.

**Figure 7:**
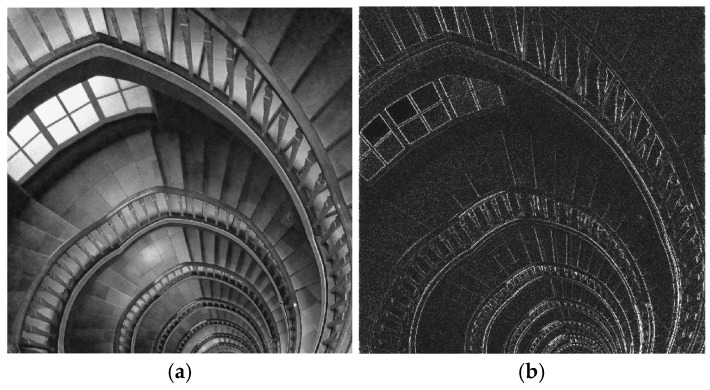

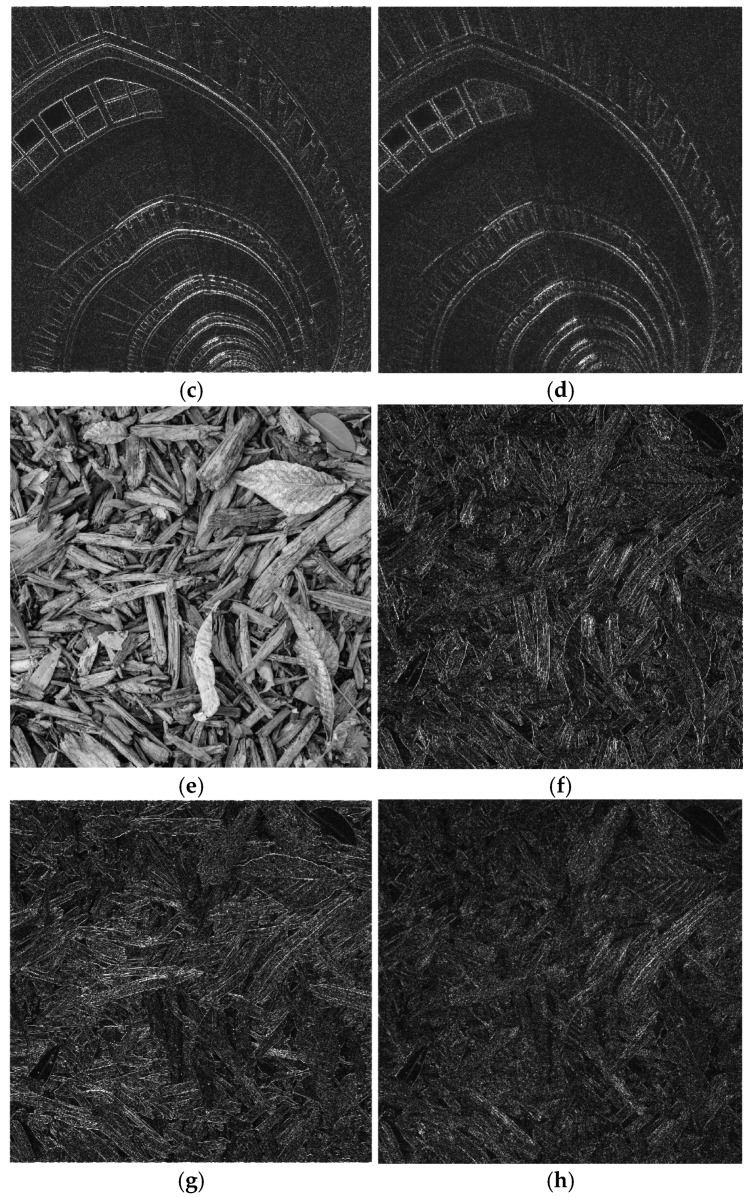
(**a**) Greyscale image “Staircase” of size 999 × 999; (**e**) greyscale image “Wood chips” of size 999 × 999; (**b**,**c**,**f**,**g**) output images resulted from the 1D FIR differentiator, applied on rows and columns, respectively; (**d**,**h**) output images resulted from the 2D FIR differentiator.

The third example in Figure 7a uses a test image (“Staircase”, 999 × 999 pixels) showing a spiral staircase viewed from above. In this image, as in the previous one, the perspective effect makes the details (i.e., stairs, handrails) at lower floors appear smaller; therefore, this image also has progressively coarse to fine details, being suitable for showing the performance of the differentiator. The differentiated images are shown in Figure 7b–d.

The fourth grayscale image “Wood chips” (999 × 999 pixels) shows a layer of wood chips of random sizes and positions, actually forming a specific texture, also containing coarser and finer details. The filtered images are given in Figure 7f–h.

The observations regarding the images resulted at the output of the 1D and 2D differentiators on the binary images in Figure 5a,e remain valid also for the grayscale images used in Figure 6 and Figure 7. The four real-life images used are cropped, grayscale versions of free stock photos available on the Pexels webpage.

It must be mentioned that these simulation results only show the elementary performance of the designed differentiator. As is well known, the edge-detection problem is a relatively complex one, and there are many well-established techniques to solve this quite difficult issue belonging to the domain of image processing.

When applying a 2D differentiator, the objects having only edges parallel to axes will be completely erased, as can be seen from the output images in Figure 5d,h. This effect is not due to separability in the frequency domain but is, rather, a principial issue due to the expression of the 2D differentiator frequency response itself, as given by (1). This is easily explained in the frequency domain. It is known that the spectrum of a straight line with a given orientation in an image also looks like a straight line in the frequency domain (but orthogonal to the line itself). From the shape of the frequency response of the 2D differentiator shown in Figure 4b, it can be seen that it is zero along both frequency axes, $\omega_{1}$ and $\omega_{2}$; this is also shown by the frequency response $H_{2D}(\omega_{1},\omega_{2})=-\omega_{1}\cdot \omega_{2}$: Along the axis $\omega_{1}$, we have $\omega_{2}=0$, while along the axis $\omega_{2}$, $\omega_{1}=0$, so in both cases, $H_{2D}(\omega_{1},\omega_{2})=0$. Since the objects with edges parallel to the axes have the spectrum made up of lines oriented along the frequency axes $\omega_{1}$ and $\omega_{2}$, these spectra are multiplied with zero when the 2D differentiator is applied, so the corresponding objects are entirely erased. Therefore, this is the effect of the 2D differentiator, regardless of the implementation (separable or nonseparable), since it is described by the same expression (1).

**Figure 8:**
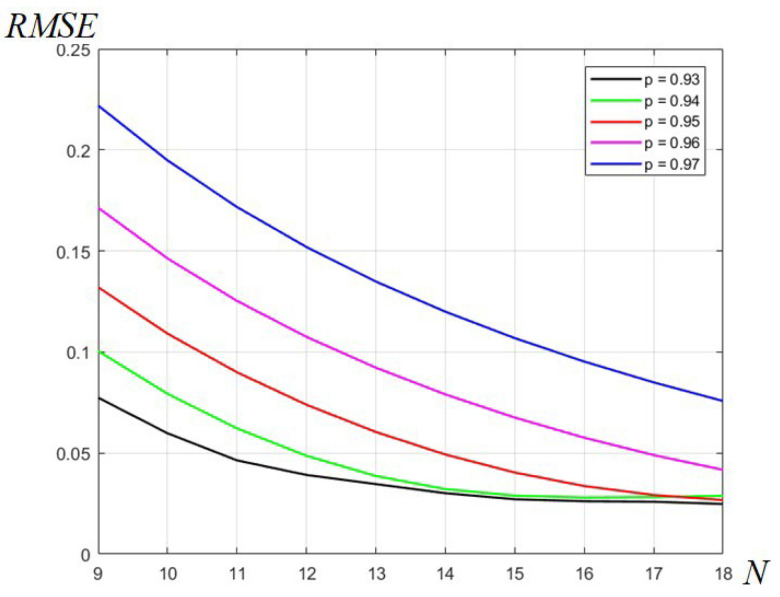
Plot of distortion error evaluated using RMSE for various orders *N* and values of parameter *p*.

As shown in the main section, the parameter *p* was introduced to attenuate the marginal oscillations due to the Gibbs effect, which appears in the Fourier series given by (3), in which the generating pulse has abrupt transitions at the limits of frequency interval, namely, −π and π. In the presented design example, the parameter value p=0.94 was used in the Fourier series given by (4), which leads to the frequency response of the 1D differentiator given by (5). This is a factored trigonometric polynomial of order N = 18 that can be directly implemented, since it can be regarded as a discrete Fourier transform of a discrete sequence, which is the FIR filter impulse response. The choice of the value *p* determines the level of distortions, mainly represented by the Gibbs oscillations, but also the marginal error represented by the finite slope that results of value p/(1−p), in our case, 15.66. Therefore, smaller values of *p* would lead to smaller oscillations for a given order, or conversely, a lower order for an allowed distortion level due to ripple. However, for smaller values of *p* (for instance, *p* = 0.9), the marginal slope decreases and the approximation will also have a smaller slope, which would cause a distortion at the margins of the interval (−π, π). In order to evaluate the distortions of a given approximation, we will use the well-known measure RMSE (root mean square error). While this measure is commonly used in statistics, it can be used in our case to evaluate the error between the approximation and the original linear function that describes the ideal differentiator. The expression of RMSE for our purpose will be the following:

(12) $$RMSE=\sqrt{\frac{1}{N}\cdot \sum_{i=1}^{N}{\left(H_{D}(\omega_{i})-\omega_{i}\right)}^{2}}$$

where *N* is the number of points on the frequency interval (−π, π), *i* is the current point (i=1 … N), $\omega_{i}$ is the frequency value and also the value of the ideal differentiator frequency response at point *i*, and $H_{D}(\omega_{i})$ is the value of the differentiator approximation at frequency $\omega_{i}$. We will calculate the distortion using RMSE in *N* = 1000 points, for the filter order *N* from 9 to 18, and for the values of parameter *p* from 0.93 to 0.97, in five steps, each of 0.1. The plots of RMSE for these values of *p* are shown in Figure 8, and the plot colors are assigned according to the attached legend. It can be noticed from this plot that the smallest RMSE values, corresponding to smallest distortions, are attained for the order *N* = 18 and parameter *p* of values 0.93, 0.94 and 0.95, for which the corresponding plots (in black, green and red) are very close. In the given design example, as mentioned, we used the filter order *N* = 18 and value *p* = 0.94.

## 4. Efficient Implementation of the 2D Separable FIR Differentiator Using Polyphase Technique

In papers like [34,35,36,37], various methods are employed to obtain separable 2D filters, due to the major advantage of efficient implementation with a significantly lower arithmetic complexity. The 2D differentiator approached in this work is inherently separable, as expressed in (1) and (2), and this feature is exploited in our design procedure so as to obtain a very computationally efficient differentiator, which is, in essence, a particular FIR filter.

If the filtering were implemented using a direct 2D convolution, the arithmetic complexity of the required computation would be prohibitive. The amount of data used by 2D filters in image and video processing applications is usually quite large and special measures have to be taken, especially in real-time processing. The high-speed implementation of 2D FIR filters used in such real-time applications is a challenging task.

In [41,42], such an implementation of separable filters using the well-known row–column procedure is employed. A direct approach involves (N/2) times more computation (where N is the row and column size of the 2D filter) compared with the row–column approach for a separable 2D filter.

**Figure 9:**
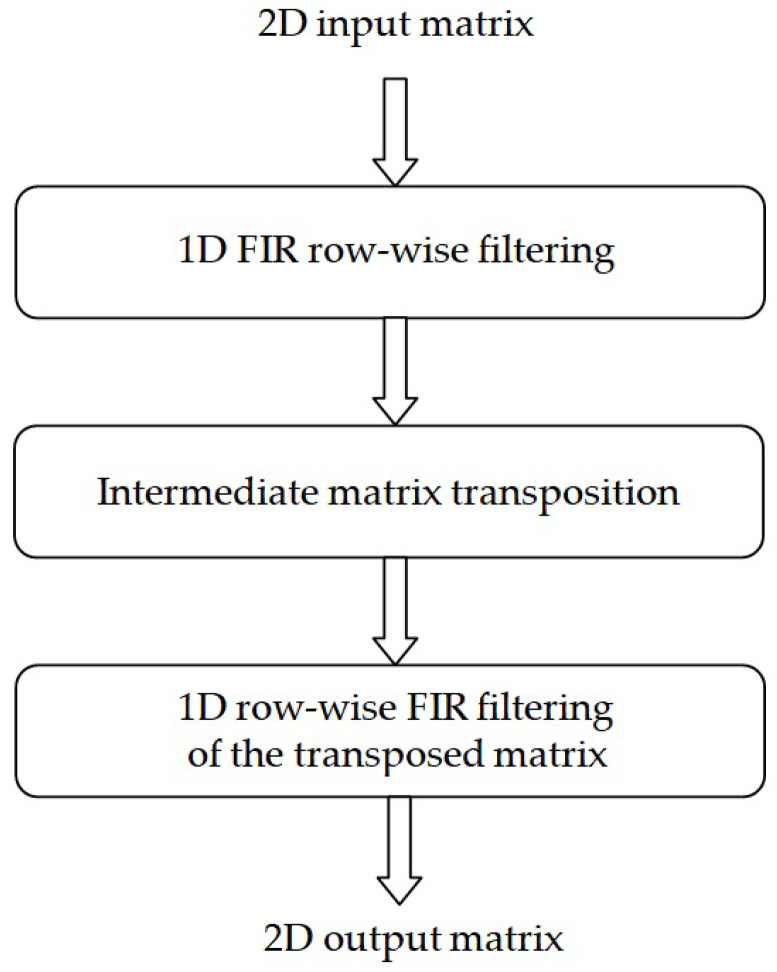
Illustration of the row–column approach [41].

The block diagram in Figure 9 illustrates the well-known row–column procedure [41].

In the row–column approach, the 1D filter is first applied to each row of the input image matrix and, subsequently, the resulting matrix is transposed using a transposition memory; then, the same 1D filter is applied again to each row of the transposed matrix, as in the following equation:

(13) $$Y=H_{1}X_{H_{2}}^{T}=H_{1}{\left(H_{2}X^{T}\right)}^{T}$$

In this section, an efficient implementation with a highly parallel structure and low computational complexity is achieved at system level for the designed separable 2D FIR differentiator, using a row–column decomposition and employing a polyphase structure and sub-expression sharing. These combined techniques achieve a 2D filtering task (in our case, a 2D differentiation) with a significantly lower arithmetic complexity and lead to a parallel implementation using block processing.

As a first step, using a sub-expression sharing technique, we developed an algorithm which achieves 1D filtering with a kernel of size 1 × 6, as follows. To perform this task, the kernel of the 1D filter resulted from the design and the input image to be processed are first decimated by a factor of 3; after this, a polyphase filtering technique is used. The main reason for developing this polyphase filtering algorithm applied to 1D FIR filters (in our case, the differentiator) was to lessen the arithmetic complexity and, in the same measure, to speed up the calculation using a parallel architecture. It is well known that performing a direct 1D filtering operation involves a high degree of redundancy in computation, caused by overlapping blocks of input data; by eliminating a large amount of these unnecessary, redundant operations, a significant and beneficial decrease in the arithmetic complexity of the system will be achieved, using a sub-expression technique.

In this polyphase technique, a decimation by factor 3 of the filter kernel will be made. Correspondingly, we also performed a decimation by factor 3 of the input image.

Combining the block processing and polyphase decomposition techniques described before, the following algorithm has been elaborated for the efficient implementation of the obtained 2D FIR filter by decomposing it into two separate, consecutive 1D filtering operations. Here, we will refer to the differentiator more generally as a filter.

The 1D FIR differentiator, which can be regarded as a particular FIR filter, is described by Equation (14):

(14) $$Y=\left[\begin{array}{c} Y_{0}\\ Y_{1}\\ Y_{2}\\ Y_{3}\\ Y_{4}\\ Y_{5} \end{array}\right]=\left[\begin{array}{ccccccccccccccccccccc} 1 & 1 & 1 & 1 & 1 & 1 & 0 & 0 & 0 & 0 & 0 & 0 & 0 & 0 & 0 & 0 & 0 & 0 & 0 & 0 & 0\\ 0 & 1 & 0 & 0 & 0 & 0 & 1 & 1 & 1 & 1 & 1 & 0 & 0 & 0 & 0 & 0 & 0 & 0 & 0 & 0 & 0\\ 0 & 0 & 1 & 0 & 0 & 0 & 0 & 0 & 0 & 0 & 0 & 1 & 1 & 1 & 1 & 0 & 0 & 0 & 0 & 0 & 0\\ 0 & 0 & 0 & 1 & 0 & 0 & 0 & 0 & 0 & 0 & 0 & 0 & 1 & 0 & 0 & 1 & 1 & 1 & 0 & 0 & 0\\ 0 & 0 & 0 & 0 & 1 & 0 & 0 & 0 & 0 & 0 & 0 & 0 & 0 & 1 & 0 & 0 & 1 & 0 & 1 & 1 & 0\\ 0 & 0 & 0 & 0 & 0 & 1 & 0 & 0 & 0 & 0 & 0 & 0 & 0 & 0 & 1 & 0 & 0 & 1 & 0 & 1 & 1 \end{array}\right]diag\left(P\cdot \left[\begin{array}{c} h_{0}\\ h_{1}\\ h_{2}\\ h_{3}\\ h_{4}\\ h_{5} \end{array}\right]\right)\cdot Q\cdot \left[\begin{array}{c} x_{0}\\ x_{1}\\ x_{2}\\ x_{3}\\ x_{4}\\ x_{5} \end{array}\right]$$

where the matrices P and Q are displayed below:

(15) $$P=\left[\begin{array}{cccccc} 0 & 0 & 0 & 0 & 0 & 1\\ 0 & 0 & 0 & 0 & 1 & 1\\ 0 & 0 & 0 & 1 & 0 & 1\\ 0 & 0 & 1 & 0 & 0 & 1\\ 0 & 1 & 0 & 0 & 0 & 1\\ 1 & 0 & 0 & 0 & 0 & 1\\ 0 & 0 & 0 & 0 & 1 & 0\\ 0 & 0 & 0 & 1 & 1 & 0\\ 0 & 0 & 1 & 0 & 1 & 0\\ 0 & 1 & 0 & 0 & 1 & 0\\ 1 & 0 & 0 & 0 & 1 & 0\\ 0 & 0 & 0 & 1 & 0 & 0\\ 0 & 0 & 1 & 1 & 0 & 0\\ 0 & 1 & 0 & 1 & 0 & 0\\ 1 & 0 & 0 & 1 & 0 & 0\\ 0 & 0 & 1 & 0 & 0 & 0\\ 0 & 1 & 1 & 0 & 0 & 0\\ 1 & 0 & 1 & 0 & 0 & 0\\ 0 & 1 & 0 & 0 & 0 & 0\\ 1 & 1 & 0 & 0 & 0 & 0\\ 1 & 0 & 0 & 0 & 0 & 0 \end{array}\right]Q=\left[\begin{array}{cccccc} z^{-5}-1 & -z^{-4} & -z^{-3} & -z^{-2} & z^{-1} & 0\\ 0 & z^{-4} & 0 & 0 & 0 & 0\\ 0 & 0 & z^{-3} & 0 & 0 & 0\\ 0 & 0 & 0 & z^{-2} & 0 & 0\\ 0 & 0 & 0 & 0 & z^{-1} & 0\\ 1 & 0 & 0 & 0 & 0 & 0\\ -1 & -z^{-4}-1 & z^{-3} & -z^{-2} & -z^{-1} & 0\\ 0 & 0 & 0 & z^{-2} & 0 & 0\\ 0 & 0 & 0 & 0 & z^{-1} & 0\\ 1 & 0 & 0 & 0 & 0 & 0\\ 0 & 1 & 0 & 0 & 0 & 0\\ -1 & -1 & -z^{-3}-1 & -z^{-2} & z^{-1} & 0\\ 1 & 0 & 0 & 0 & 0 & 0\\ 0 & 1 & 0 & 0 & 0 & 0\\ 0 & 0 & 1 & 0 & 0 & 0\\ -1 & 1 & -1 & -z^{-2}-1 & -z^{-1} & 0\\ 0 & 1 & 0 & 0 & 0 & 0\\ 0 & 0 & 1 & 0 & 0 & 0\\ -1 & -1 & -1 & 1 & -z^{-1}-1 & 0\\ 0 & 0 & 0 & 0 & 1 & 0\\ -1 & -1 & -1 & -1 & -1 & 1 \end{array}\right]$$

Using the polyphase decomposition, we obtain a 1D FIR filter given by Equation (16):

(16) $$Y=\left[\begin{array}{c} Y_{0}\\ Y_{1}\\ Y_{2}\\ Y_{3}\\ Y_{4}\\ Y_{5} \end{array}\right]=\left[\begin{array}{ccccccccccccccccccccc} 1 & 1 & 1 & 1 & 1 & 1 & 0 & 0 & 0 & 0 & 0 & 0 & 0 & 0 & 0 & 0 & 0 & 0 & 0 & 0 & 0\\ 0 & 1 & 0 & 0 & 0 & 0 & 1 & 1 & 1 & 1 & 1 & 0 & 0 & 0 & 0 & 0 & 0 & 0 & 0 & 0 & 0\\ 0 & 0 & 1 & 0 & 0 & 0 & 0 & 0 & 0 & 0 & 0 & 1 & 1 & 1 & 1 & 0 & 0 & 0 & 0 & 0 & 0\\ 0 & 0 & 0 & 1 & 0 & 0 & 0 & 0 & 0 & 0 & 0 & 0 & 1 & 0 & 0 & 1 & 1 & 1 & 0 & 0 & 0\\ 0 & 0 & 0 & 0 & 1 & 0 & 0 & 0 & 0 & 0 & 0 & 0 & 0 & 1 & 0 & 0 & 1 & 0 & 1 & 1 & 0\\ 0 & 0 & 0 & 0 & 0 & 1 & 0 & 0 & 0 & 0 & 0 & 0 & 0 & 0 & 1 & 0 & 0 & 1 & 0 & 1 & 1 \end{array}\right]diag\left(P\cdot \left[\begin{array}{c} H_{0}\\ H_{1}\\ H_{2}\\ H_{3}\\ H_{4}\\ H_{5} \end{array}\right]\right)\cdot Q\cdot \left[\begin{array}{c} X_{0}\\ X_{1}\\ X_{2}\\ X_{3}\\ X_{4}\\ X_{5} \end{array}\right]$$

Thus, for the particular case in which the kernel matrix for 1D FIR filter is of size 1 × 18 and using the decimation factor of 3 for the input image, the vectors $H_{0}$, $H_{1}$, $H_{2}$, $H_{3}$, $H_{4}$ and $H_{5}$ will have the following forms:

(17) $$\begin{array}{l} H_{0}=[h_{0}\;h_{6},h_{12}]\;;\;H_{1}=[h_{1}\;h_{7},h_{13}]\;;\;H_{2}=[h_{2}\;h_{8},h_{14}]\;;\;\\ H_{3}=[h_{3}\;h_{9},h_{15}]\;;\;H_{4}=[h_{4}\;h_{10},h_{16}]\;;\;H_{5}=[h_{5}\;h_{11},h_{17}] \end{array}$$

For the particular case in which the input matrix for 1D FIR filter is of size 1 × 18 and the decimation factor is 3, the input vectors $X_{0}$, $X_{1}$, $X_{2}$, $X_{3}$, $X_{4}$ and $X_{5}$ will have the following forms:

(18) $$\begin{array}{l} X_{0}=[\begin{array}{ccc} x_{0} & x_{6} & x_{12} \end{array}]\;;\;X_{1}=[\begin{array}{ccc} x_{1} & x_{7} & x_{13} \end{array}]\;;\;X_{2}=[\begin{array}{ccc} x_{2} & x_{8} & x_{14} \end{array}]\;;\;\\ X_{3}=[\begin{array}{ccc} x_{3} & x_{9} & x_{15} \end{array}]\;;\;X_{4}=[\begin{array}{ccc} x_{4} & x_{10} & x_{16} \end{array}]\;;\;X_{5}=[\begin{array}{ccc} x_{5} & x_{11} & x_{17} \end{array}] \end{array}$$

The proposed polyphase decomposition technique, applied here in a particular case, namely, for the specified length of the 1D differentiator, can be readily extended to larger filters; therefore, in this respect, this approach is scalable. For example, if the 2D FIR filter size is 36 × 36, we simply replace the vectors $H_{0}$, …, $H_{5}$ and $X_{0}$, …, $X_{5}$, respectively (containing three elements), with the vectors $H_{0}$, …, $H_{5}$ and $X_{0}$, …, $X_{5}$, containing six elements, without any other modification.

This implementation approach leads to a highly parallel structure and significantly decreases the arithmetic complexity. To put in evidence its efficiency, the elaborated filtering technique can be compared with the direct convolution operation between an image and the filter kernel of large size, as regards computational requirements. Thus, the filtering operation of an image of size M×N pixels, performed in the traditional manner, using an FIR filter with a kernel of size m×n, requires a 2D convolution between a m×n matrix and a M×N matrix. This means that the filter kernel slides horizontally and vertically along the image, so for each one of the M×N image pixels, m×n multiplications are required; therefore, the entire 2D filtering operation is of the order of complexity O(MNmn). Moreover, the total number of additions would be $\left(N+n^{2})(M+m^{2}\right)$ in direct convolution.

Regarding the evaluation of arithmetic complexity, the proposed polyphase implementation of the 2D FIR differentiator requires only 63 multiplications and additions in the 21 inner products, 30 additions in the post-processing stage and 30 additions and some shift operations in the preprocessing stage for each 1D FIR differentiator, applied to each image block of size 18 × 18 pixels. There are MN/(18 × 18) such blocks in an image of size MN pixels.

## 5. Discussion

One major benefit of the presented analytical design procedure is that the frequency response of the FIR digital differentiator is derived without using complicated numerical optimization techniques. As the ideal differentiator is accurately approximated using the Fourier series or the very efficient Chebyshev series, the designed differentiator will be very close to its optimal version. The transfer function of the resulted 2D differentiator in both versions will be a product of two 1D transfer functions, expressed in the two frequency variables, $\omega_{1}$ and $\omega_{2}$. The proposed design leads to digital differentiators with a relatively low order and accurate frequency response, which is separable along the two axes and results in being directly decomposed into second-order factors, also allowing for a sequential implementation.

Both approximations lead to efficient differentiators. The first one, based on Fourier series, is more flexible and easier to obtain, since it has an analytic form given by (4); for any given order required by an imposed error, we get directly a trigonometric polynomial which is factored as in (5) or expressed in variable *z* as in (10). The second version, relying on Chebyshev series, is somewhat more laborious, as it requires the change in variable (6) and the use of a symbolic calculation software to derive the Chebyshev series. However, for the same order, this method gives a slightly more linear differentiator, as shown in Figure 3. Of course, for less demanding applications, where perfect linearity is not necessary, the order of approximation can be reduced, at the expense of a higher degree of distortion.

Regarding the arithmetic complexity of the proposed FIR differentiator, it is comparable or superior to other solutions available in the literature. This differentiator designed through analytical approach has an accurate shape and a relatively low order.

Due to the wide variety of methods and very different approaches existing in the literature for the design of digital differentiators, a strict comparison in terms of performance between the method proposed here and other techniques from various works is difficult to make. However, it can be noticed that the 1D differentiator designed in this paper, as a component of the separable 2D differentiator, has a remarkable linearity and negligible distortions, having superior performances as compared to some designs found in the literature. For instance, the FIR differentiators proposed in [20,25,26], with edge detection applications, are indeed of a lower order but their frequency characteristics diverge significantly from the perfectly linear characteristic of the ideal prototype, such that the frequency range on which they perform a precise differentiation is more limited. As shown in the main text, the differentiator proposed in this work, in both versions (with Fourier and Chebyshev series approximation), operates correctly on the entire frequency range, with minimal distortions, mainly towards the margins of the interval. These distortions were evaluated using, as a measure, the RMSE, and the choice of parameter *p* for the chosen filter length is nearly optimal. Our 1D differentiator has a higher order but it is very similar in shape to its ideal version; therefore, its precision in various image processing tasks should be superior. Moreover, through our efficient implementation technique using polyphase and block filtering, the higher order should not be a critical issue, since the parallel processing and reduced overall arithmetic complexity significantly lower the computation time. Another advantage of our solution is that the designed differentiator is essentially zero-phase and does not produce phase distortions in the processed image.

As a remark, the Chebyshev series approach is a little more laborious than the Fourier series version, since the drawback of Chebyshev series is that its coefficients cannot be expressed analytically, but only obtained numerically for given specifications (function, interval and order of approximation). By comparison, the Fourier series coefficients are obtained analytically, as show Equations (3) and (4). In this respect, the Chebyshev series approach may be regarded as more computationally demanding. Anyway, symbolic software like Maple provides the Chebyshev expansion in a matter of seconds; therefore, this drawback is quite irrelevant. Moreover, unlike the case when we need to design various filters with arbitrary specifications, the 2D differentiator is unique, in the sense that it is given by the transfer function (1), which has no parameters. Therefore, this calculation is performed only once, for a specified order or precision, as shown in our paper.

In the general case, however, the analytical design method of 2D filters using Chebyshev series and frequency transformations applied on 1D prototypes is more convenient compared to global numerical optimization techniques, since the imposed specifications (bandwidth, selectivity, etc.) appear explicitly as parameters in the designed 2D filter frequency response [40]. Thus, when changing the specifications, they are simply substituted in the 2D filter frequency response that results directly; there is no need to resume the entire design process every time from the start.

It is well known that differentiators, when applied both on continuous and discrete time signals, as well as on images, tend to amplify the high frequencies, since the frequency response magnitude is proportional to the frequency. Therefore, for images with a high level of noise, it is to be expected that noise will affect the output image, which is quite challenging in tasks like edge detection. Various types of noise (Gaussian, salt-and-pepper, speckle noise, etc.) may blur the object edges or create false ones, and reduce the contrast, thus affecting the quality of edge detection. Solutions to this problem are well known, such as Gaussian smoothing, median filtering and bilateral filtering, which replace each pixel value with a weighted average of its neighbors, thus reducing noise while preserving and enhancing edges. Anyway, as already mentioned before, for edge-detection tasks, other operators are generally preferred instead of the elementary differentiator. The issue of using the designed differentiator in more complex applications, for instance, in processing multi-dimensional signals beyond images, remains to be investigated in future work.

The advantage of the Chebyshev series is that it yields a more uniform approximation, with constant error along the entire specified interval, while the approximation using Fourier series tends to present oscillations (ripple) due to the Gibbs effect, especially in the vicinity of abrupt variations, as is clearly visible in Figure 1b,c and Figure 2b. However, in our case, for the designed differentiator of order N = 18 and the chosen value for parameter p, both approaches give practically very linear approximations almost on the entire frequency range.

Regarding the implementation issue, the extension to the 3D domain is not very easy; in further research on this topic, we will also investigate this possible extension, which may have some degree of difficulty, and may require the use of elements of tensor calculus. This may be the subject for a future work on an analytical approach to the design of 3D FIR filters, in particular, differentiators.

For larger data sets, we can divide the image of size M × N into blocks of a larger size, as has been shown above, and using parallelism with a reduced arithmetic complexity, we can process these large data sets in real-time with a significantly smaller processing time as compared with the traditional approach.

Further research on the topic will also investigate how the proposed digital differentiators can be improved regarding arithmetic complexity, finding various implementation architectures and also the possibility to extend this design technique to other types of such filters.

## 6. Conclusions

The proposed analytical design achieved in the frequency domain results in a very efficient and accurate 2D FIR differentiator, which is separable into two 1D differentiators along the two axes of the frequency plane. This design method is relatively simple and is based on Fourier series or Chebyshev series approximation; no other numerical optimization is required. The frequency response of the designed 2D FIR differentiator results in a very accurate shape, with good linearity and negligible distortions. Depending on the required accuracy, the order of approximation can be higher or lower and, therefore, the computational complexity of the differentiators will vary, but in general, they are very efficient. The implementation based on a polyphase and block filtering approach leads to a structure with low arithmetic complexity.

## Figures and Tables

**Table 1:** FIR differentiator coefficients.

Order	Coefficient	Order	Coefficient	Order	Coefficient
18	−0.0057746	6	0.0840918	−6	−0.0840918
17	0.0089107	5	−0.1273239	−7	0.0525547
16	−0.0118254	4	0.1892063	−8	−0.0292339
15	0.0141471	3	−0.2861314	−9	0.0121436
14	−0.0154454	2	0.4677452	−10	0.0000002
13	0.0152378	1	−0.9836315	−11	−0.0081292
12	−0.0129929	0	0	−12	0.0129929
11	0.0081292	−1	0.9836315	−13	−0.0152378
10	−0.0000002	−2	−0.4677452	−14	0.0154454
9	−0.0121436	−3	0.2861314	−15	−0.0141471
8	0.0292339	−4	−0.1892063	−16	0.0118254
7	−0.0525547	−5	0.1273239	−17	−0.0089107
				−18	0.0057746

## Data Availability

The datasets (images) analysed during the current study are available in the OSF repository, at the following link: https://osf.io/8prdf (accessed on 29 November 2024).
